# Evaluation of association between airway hyperresponsiveness, asthma control test, and asthma therapy assessment questionnaire in asthmatic children

**DOI:** 10.1186/2049-6958-8-48

**Published:** 2013-07-23

**Authors:** Daniele Rapino, Marina Attanasi, Nicola P Consilvio, Alessandra Scaparrotta, Anna Cingolani, Marzia Cerasa, Angelika Mohn, Sabrina Di Pillo, Francesco Chiarelli

**Affiliations:** 1 Allergy and Respiratory Unit, Department of Pediatrics, G. D’Annunzio University of Chieti, Via Dei Vestini 5, Chieti 66013, Italy; 2 Department of Pediatrics, G. D’Annunzio University of Chieti, Via Dei Vestini 5, Chieti 66013, Italy

**Keywords:** Asthma, Asthma control, ACT, ATAQ, Airways hyperresponsiveness

## Abstract

**Background:**

Achieving asthma control is a major challenge in children, otherwise symptoms perception remain poor especially at this age. The aim of this study is to evaluate the relationship between Asthma Control Test (ACT^TM^), Asthma Therapy Assessment Questionnaire (ATAQ^TM^) and exercise-induced bronchospasm (EIB).

**Methods:**

We studied 80 asthmatic children. Airways hyperresponsiveness (AHR) was assessed by exercise-induced bronchospasm (Balke Protocol). Asthma control was evaluated using two questionnaires in all subjects: ACT (composed by Childhood-ACT and ACT) and ATAQ. In addition the use of short acting beta 2 agonist agents (SABAs) was assessed for each patient. Non-parametric variables were compared by Chi Square Test. Binomial logistic regression was performed to estimate the two questionnaires Odds Ratio (OR) in finding AHR.

**Results:**

We have found that ATAQ has a sensitivity and a specificity of 0.72 and 0.45 respectively; instead, ACT has a sensitivity and a specificity of 0.5 and 0.39 respectively in evaluating AHR. Patients with uncontrolled asthma according to ATAQ revealed a significant higher percentage of AHR compared with ACT (72% vs 50%, p < 0.01).

Confirming this finding, patients declaring uncontrolled asthma to ATAQ have a significantly higher percentage (34%) of frequent SABAs use than the group with uncontrolled asthma to ACT (21%) (p <0.01).

Binomial logistic regression shows how a test revealing uncontrolled asthma is associated with the increasing odds of having AHR according to ATAQ (OR = 3.8, p = 0.05), not to ACT (OR = 0.2, p = 0.1).

**Conclusions:**

Our results show that ATAQ reflects AHR and asthma control better than ACT. Children with uncontrolled asthma according to ATAQ have higher odds of having AHR and use of rescue medications (SABAs) compared to patients declaring uncontrolled asthma according to ACT. However both questionnaires are not sufficient alone to fully evaluate asthma control in children and it is always necessary to perform functional tests and investigate patients lifestyle, drug use and other important data that a simple questionnaire is not able to point out

## Background

Asthma prevalence increased during the last decade. Compared with adults, children had higher rates for asthma primary care and Emergency Department visits, but similar hospitalization rates, and lower death rates [1].

In spite of recent advances in the detection and treatment of this pathology, asthma remains a cause of significant morbidity and constitutes an economic burden for the society.

Asthma is one of the major causes of emergency visits per year [2] and is the third leading cause of preventable hospitalization [3].

A divergence between perception of asthma control in patients and asthma control according to guidelines is indubitably present. In a study on several hundred asthmatic patients, despite the fact that 90% of patients were satisfied with their asthma treatment results, just 50% of them had an acceptable asthma control according to guidelines [4].

Asthma management and, in particular, early detection of asthma symptoms remain a crucial objective of clinical control for this disease.

Several asthma control questionnaires promising better evaluation of the asthmatic patients have been validated but the information regarding their differences is at present scarce in the literature on this subject.

In this study we assessed Asthma Control Test (ACT™), a validated auto-administered questionnaire that ascertains clinical control in asthmatic patients by few simple questions [5,6] and Asthma Therapy Assessment Questionnaire (ATAQ™), a short administrable questionnaire developed to assist clinicians and health plans to identify children at risk for adverse outcomes of asthma [7].

In addition all subjects performed an incremental treadmill exercise test following a standard Balke protocol [8,9]. Stress tests have been considered in the “PRACTALL Consensus” as additional tools to evaluate the efficacy of asthma therapy and asthma control [10]. The aim of this study was to evaluate the correlations between these two questionnaires and a standardized stress test. As a second outcome ATAQ and ACT were compared in order to highlight the differences between them and underline when and why one of these asthma control questionnaires should be used.

## Methods

### Subjects

Eighty children (53 boys and 27 females) were recruited from the Allergy, Pulmonology and Immunology Division of Pediatric Department, University of Chieti, Italy. All patients were previously diagnosed with asthma without any other respiratory illness. Study population is shown in Table 1 and Table 2.

**Table 1:** Characteristics of study population

**Variable**	
Sex M/F	53/27
Age (years, mean ± standard deviation, SD)	9.6 ± 3.3
Beta 2 agent use +/−	45/35
AHR (Balke protocol) +/−	46/34
ACT +/−	12/68
ATAQ +/−	22/58

**Table 2:** Basal spirometry

**Basal spirometry**	**Mean ± SD**
FEV_1_%	109 ± 11
FVC%	99 ± 12
FEF_75_%	111 ± 31
FEF_50_%	100 ± 18
FEF_25_%	99 ± 12

Asthma was classified according to Global INitiative for Asthma guidelines [11] as intermittent, mild persistent, moderate persistent, or severe persistent asthma. The diagnosis was established by a single pediatric respiratory physician according to American Thoracic Society European Respiratory Society (ATS/ERS) criteria [12] on the basis of a clinical history of repeated episodes of coughing, dyspnea, and wheezing.

### Lung function evaluation

Lung function evaluation was performed by flow/volume curves in the standing position with nose-clip until two consecutive technically acceptable curves were achieved, according to ATS/ERS guidelines [9]. The main spirometric parameters included forced expiratory volume in 1 second (FEV_1_), forced vital capacity (FVC), the ratio FEV_1_/FVC, and forced expiratory flows between 25% and 75% of the FVC [10]. All lung function parameters were expressed as a percentage of theoretical values for age, gender, and height according to Quanjer et al [13].

### Airways hyperresponsiveness (AHR) assessment

The subjects performed an incremental treadmill exercise test following a standard Balke protocol [8,9]; as suggested for clinical stress test in children, temperature was in the range of 20–24°C with a relative humidity between 50% and 60%. All patients reached a heart rate higher than 80% of the predicted maximum (calculated as 220 less the age in years), according to ATS/ERS recommendations for exercise challenge test in children [14]. Spirometry was measured at 1, 3, 5, 10, and 20 minutes after the exercise. The patients performed two **s**pirometries every minute; the best of the two performed curves was selected as the representative value at each interval, but the differences between the two values of FEV_1_ had to be <5%. A bronchial obstruction response to exercise was considered positive when the FEV_1_ reduction was greater than 10% from baseline [15]. Exercise response was defined as the greatest fall in FEV_1_ expressed as a percentage of the baseline values.

### Asthma control

Levels of asthma control were evaluated by two questionnaires in all subjects: 1) the ACT (the 5 questions ACT for children over 12 years old and the 7 questions Childhood ACT for the children from 4 to 11 years old) and 2) the ATAQ (we used the 7 questions version validated for children and teens from 5 to 17 years old).

Because these are considered auto-administrable questionnaire they were filled out by children and parents: ACT (>12 years) was completed by children themselves, ACT (4–11 years) by children and parents, while ATAQ was completed entirely by the parents.

Questionnaires were filled in before patients performed any other procedures; in addition physicians were available to assist the completion of the questionnaires if the parents or children had some questions about semantic or conceptual understanding.

Patients with a score under 19 for ACT and above 1 for ATAQ according to the literature [5,7] (Tables 3, 4, 5) were considered having uncontrolled asthma.

**Table 3:** ASTHMA CONTROL TESTfor children over 12 years of age

1.	In the past 4 weeks, how much of time did your asthma keep you from getting as much done at work, school or at home?
2.	During the past 4 weeks, how often have you had shortness of breath?
3.	During the past 4 weeks, how often did your asthma symptoms (wheezing, coughing, shortness of breath, chest tightness or pain) wake you up at night or earlier than usual in the morning?
4.	During the past 4 weeks, how often have you used your rescue inhaler or nebulizer medication (such as albuterol)?
5.	How would you rate your asthma control during the past 4 weeks?

**Table 4:** ASTHMA CONTROL TEST (children from 4 to 11 years of age)

1.	How is your asthma today?
2.	How much a problem is your asthma when you run, exercise or play sports?
3.	Do you cough because of your asthma?
4.	Do you wake up during the night because of your asthma?
5.	During the last 4 weeks, how many days did your child have any daytime asthma symptoms?
6.	During the last 4 weeks, how many days did your child wheeze during the day because of asthma?
7.	During the last 4 weeks, how many days did your child wake up during the night because of asthma?

**Table 5:** **ATAQ questionnaire (for children** &**teens, from 5 to 17 years old)**

	In the past 4 weeks, did your child:
1.	Have wheezing or difficulty breathing when exercising?
2.	Have wheezing during the day when not exercising?
3.	Wake up at night with wheezing or difficult breathing?
4.	Miss days of school because of his/her asthma?
5.	Miss any daily activities (such as playing, going to a friend’s house, or any family activity) because of asthma?
6.	Does your child use an inhaler or a nebulizer for quick relief from asthma symptoms? (multiple choice question)
7.	Do you believe that your child’s asthma was well controlled in the past 4 weeks?

### Beta 2 agonist use

During the clinical evaluation the physician collected information about the use of SABAs and children were stratified if they need to use this class of drugs more than 2 times during the last month (frequent SABAs use).

### Statistical analysis

The two groups (ATAQ and ACT) have been studied for non-parametric variables by Chi Square Test. For each questionnaire it was obtained the sensitivity and specificity in achieving AHR. Binomial logistic regression was performed to estimate the two questionnaire Odds Ratio (OR) in finding AHR; p < 0.05 was considered significant. SPSS (version 17.0 SPSS, Inc, Chicago, III) was used for data analysis.

### Ethical committee and informed consent

The study was approved by the ethical committee of the University of Chieti. Written informed consent was obtained by all parents and the study was performed in accordance with Helsinki Declaration (1964).

## Results

Exercise AHR was present in 60% of patients. While with ACT 85% of patients declared a good and 15% a bad asthma control, with ATAQ the well-controlled patients were reduced to 72.5% and the uncontrolled ones increased to 27.5%.

Patients with a score showing controlled asthma presented AHR in 55% and 61% respectively for ATAQ and ACT; patients with a score showing uncontrolled asthma presented AHR in 72% and 50% respectively for ATAQ and ACT (Table 6).

**Table 6:** Percentage of positive and negative exercise induced AHR tests in patients with controlled and uncontrolled asthma according to ATAQ and ACT

**Questionnaire**	**AHR positive (%)**	**AHR negative (%)**
Uncontrolled asthma (ACT)	**50**	**50**
Uncontrolled asthma (ATAQ)	**72**	**28**
Controlled asthma (ACT)	**61**	**49**
Controlled asthma (ATAQ)	**55**	**45**

Considering the international accepted cut off for ATAQ and ACT, we have found that ATAQ has a sensitivity and a specificity of 0.72 and 0.45 respectively, instead ACT has a sensitivity and a specificity of 0.5 and 0.39 respectively in evaluating AHR.

Binomial logistic regression confirmed how a test revealing uncontrolled asthma is associated with the odds of having AHR for the ATAQ (OR = 3.8, p = 0.05), not for the ACT (OR = 0.2, p = 0.1).

In fact patients with a score showing uncontrolled asthma according to ATAQ had a significantly higher percentage of AHR compared to patients with uncontrolled asthma according to ACT (72% vs 50%, p < 0.01).

Furthermore, we found that patients having uncontrolled asthma to ATAQ had a significantly higher percentage (34%) of frequent SABAs use than the group with uncontrolled asthma according to ACT (21%) (p < 0.01).

## Discussion

Despite the improvement in asthma knowledge and therapies, asthma control remains a difficult objective to achieve. Increasingly recognized is the importance of tools developed to help physicians and patients measuring asthma symptoms especially in children [16].

Many questionnaires have been developed to evaluate asthma patients, but additional studies in different populations are still required to assess their validity and clinical utility in comparison with objective tests.

ACT is an auto-administrable, easy-to-use validated questionnaire, composed of a few simple questions. These features render it very useful in screening and evaluating patients at home. ACT score is related to the specialists evaluation, and studies published in literature have shown that an improvement in FEV_1_ in two different visits is reflected by/correlates with an improvement in ACT score [17,18].

On the contrary, some studies have demonstrated that the ACT could overestimate asthma control and does not have a direct and strong correlation with lung function [19].

ATAQ consists of a form validated for all patients under the age of 18. A large study has shown that ATAQ could be useful in identifying children needing more intensive asthma management. Patients with the worst asthma control using the pediatric ATAQ questionnaire had significantly higher rates of asthma-related hospitalizations, emergency room or emergency care and doctor visits/medical appointments than those with good control [20].

ATAQ showed to correspond well with asthma control, with measures of physical health, psychosocial health, resources use, and family impact. It seems to help healthcare with asthma management plans, in fact it was shown to be significantly and positively associated with symptoms and parental satisfaction [7].

Few studies have been published about the relationships between ACT or ATAQ and AHR. In particular, no studies have been carried out to study the same population with both questionnaires and comparing the two questionnaires with a validated stress test [21,22]. This is an important feature to investigate due to the fact that stress tests have been shown to be useful in evaluating asthma control and the efficacy of asthma therapy as indicated in the PRACTALL consensus [10].

The lack of concordance between ACT results and AHR is in agreement with the literature [21,22], whereas there was no information or studies published about ATAQ.

We have found that children with uncontrolled asthma to ATAQ have a significantly higher probability of having AHR compared with patients having uncontrolled asthma with ACT.

In fact, patients with uncontrolled asthma to ATAQ presented a significantly higher percentage of positive stress tests than ACT; this finding suggests that a patient with uncontrolled asthma according to ATAQ is more likely to experience airways hyper- reactivity compared to ACT.

After evaluating ATAQ and ACT in patients with uncontrolled asthma and comparing these patient subgroups, we noted that patients with a score showing uncontrolled asthma according to ATAQ questionnaire had a significantly higher rate in beta 2 agonist agents use than patients with uncontrolled asthma according to ACT. This finding confirms how patients with a score showing uncontrolled asthma according to ATAQ more likely have AHR, but they also present higher use of rescue medication, emphasizing that there is a better correlation between ATAQ, asthma control and AHR compared to ACT. Instead, in both questionnaires, a score showing good asthma control provides no information regarding airways hyperreactivity.

Additional studies on asthma control and AHR are necessary to investigate perception of symptoms by the patients during exercise and the degree of airways hyperreactivity.

The inability of a simple questionnaire to reveal AHR could be explained because children experiencing mild asthma symptoms may consider these normal, and in this case they will not report any problem filling out the questionnaire. This consideration is confirmed in literature, in fact Panditi et al. found no correlation between asthma perception declared by children and exercise induced bronchospasm during exercise [23].

As a second explanation, asthmatic patients tend to change their lifestyle avoiding exercise, a possible trigger of bronchospasm and symptoms related to AHR; so they could not experiment asthma symptoms because they do not reach a significant exercise threshold.

Despite both ACT and ATAQ lack specificity in detecting AHR, in our study children with uncontrolled asthma to ATAQ have higher percentage of AHR compared to those with uncontrolled asthma to ACT.

ATAQ has a simple score system: all questions offer answer options as “yes, no, unsure” (Table 5) whereas the ACT has a score system ranging from 1 to 5 for each question, and the childhood ACT version is enriched with an iconography to help children in answering the questions.

ATAQ results easier to administer and more suitable as auto-administrable questionnaire, improving the power to detect AHR and asthma symptoms in children.

Moreover ATAQ has two questions investigating exercise induced symptoms, whereas ACT has no question in the version for children over 12 years old and only one question in the C-ACT for children under 12 years old (Tables 3 and 4); suggesting that the structure of the questionnaire should be reorganized to improve its specificity regarding a particular asthma feature. However, another study has shown that ACT does not improve its specificity in achieving airway hyperresponsiveness even adding in the questionnaire a sixth question specifically aimed at detecting exercise-induced asthma confirming that this questionnaire is not able to determine whether a child has AHR or not [21].

Whichever the questionnaire structure will be, it is important to obtain a good result in terms of acceptable asthma control. Another important consideration is that it is very difficult to understand how much children (and parents) are reliable in reporting symptoms or drug use. This is an important feature that has to be investigated besides improving questionnaires efficacy to evaluate asthma control.

## Conclusions

Our results demonstrate that ATAQ is more suitable than ACT to testify AHR and asthma control. Children with uncontrolled asthma according to ATAQ have higher probability of AHR and of a greater use of rescue medications (SABAs) than patients with uncontrolled asthma according to ACT. Both questionnaires alone are not sufficient to fully evaluate asthma control in children, so as functional tests, data on patients lifestyle and drug use, and other important information are always to be achieved in order to obtain a reliable evaluation.

## Abbreviations

AHR: Airway hyperresponsiveness; ACT: Asthma control test; ATAQ: Asthma therapy assessment questionnaire; SABAs: Short acting beta 2 agonist agents.

## Competing interest

All the authors report no conflicts of interest.
